# Effectiveness of Hysteroscopic Repair of Uterine Lesions in Reproductive Outcome 

**Published:** 2014-07-08

**Authors:** Atta Allah Ghahiry, Elahe Refaei Aliabadi, Ali Akbar Taherian, Aida Najafian, Mojdeh Ghasemi

**Affiliations:** 1 Department of Obstetrics and Gynecology, School of Medicine, Alzahra Hospital, Isfahan University of Medical Sciences, Isfahan, Iran; 2 Department of Obstetrics and Gynecology, School of Medicine, Hormozgan University of Medical Sciences, Bandarabas, Iran; 3 Research Office of Shahid Beheshti Hospital, Isfahan University of Medical Sciences, Isfahan, Iran

**Keywords:** Hysteroscopy, Uterus, Myomectomy, Adhesion, Abnormal Uterine Bleeding, Infertility

## Abstract

**Background:**

Small intrauterine lesions such as septum, adhesion, polyp, and submucous myoma may be of greater significance in causing implantation failure, poor reproductive performance and abnormal uterine bleeding. We studied effectiveness of therapeutic intervention through operative hysteroscopy in improvement of pregnancy outcome and cessation of abnormal uterine bleeding (AUB) in women with pregnancy and fertility problems.

**Materials and Methods:**

This prospective cohort study was performed between 2003- 2009 on 65 patients with primary or secondary infertility, recurrent abortion and structural uterine lesions reported in sonography or hysterosalpingography. After hysteroscopic metroplasty, myomectomy, adhesiolysis and polypectomy under laparoscopic guide, we evaluated reproductive outcome, early and late complications, one year after surgery.

**Results:**

Among all patients with recurrent abortion, 6 patients (75%) complete their pregnancy successfully. Our results showed that pregnancy rate after metroplasty was 58%. Reproductive outcome was poor after myomectomy and adhesiolysis. Abnormal uterine bleeding was improved in 62% of patients.

**Conclusion:**

Structural uterine lesions has been assumed to cause infertility, while several studies have shown very poor reproductive performance with high miscarriage and low term delivery rates when malformation is not treated. We show improvement in conceptional outcome and in patient’s chief complaints after hysteroscopy surgery of these anomalies.

## Introduction

Infertility and abnormal uterine bleeding due to structural lesions of uterus is the subject of many hysteroscopy procedures.

The successful pregnancy outcome depends on several factors, among which embryo qualities and intrauterine environment play major roles for the achievement and continuation of pregnancy (1). Small intrauterine lesions such as septum, adhesion, polyp, and submucous myoma are likely to be considered in causing implantation failure (2). The rate of successful pregnancies after operative hysteroscopy and medical treatment of structural uterine defects increase significantly (3). It is reasonable to think that in these cases, the treatment of choice is the restoration of a normal uterine cavity (4,5). Transabdominal surgery was used as the traditional correction method of uterine anomaly. However, this procedure had several hazards including the risk of pelvic adhesions leading to subsequent infertility, prolonged hospital stay and a longer postoperative interval before conception. Hysteroscopic surgery by enabling vaginal approach provides a simple surgical procedure and a shorter hospital stay.

Abnormal uterine bleeding is responsible for more than one third of gynecologist consultation (6). Investigation of uterine cavity enables the gynecologist to offer the most appropriate therapy, while hysteroscopy allows the visualization of probable uterine source of bleeding and helps removing it in some occasion.

The purpose of this study was to determine the effectiveness of therapeutic intervention through operative hysteroscopy in improvement of pregnancy outcome and in reducing patients’ complaints in women with pregnancy and fertility problems.

## Materials and Methods

This prospective interventional cohort study was performed between 2003 and 2009 in Alzahra and Sina hospital. It included 65 patients with primary and secondary infertility, recurrent abortion and structural uterine defects reported in sonography or hysterosalpangography (HSG). They all had normal chromosomal studies. All men had normal semen analysis according to World Health Organization criteria (more than 20 million/ml sperms with total motility of greater than 40% and more than 4% normal morphology). Therefore, individuals with male factors infertility, and other cause of infertility, except structural anomaly, were excluded from the study. All patients signed their informed consent, and the study was approved by the Ethics Committee of Isfahan University of Medical Sciences. All patients had a complete history and physical examination. They also underwent routine laboratory evaluations of blood and urine. Hysteroscopy was carried out at Alzahra and Sina Hospitals, Isfahan, Iran, with general anesthesia. All laparoscopy-guided-hysteroscopies were performed in early proliferative phase by hysteroresectoscope and laparoscope (Karl Storz GmbH and Co., Tuttlingen, Germany). A 1.5% glycine solution in a 3-liters bag was used for uterine distension and irrigation. Fluid balance was very carefully monitored throughout the procedure in order to avoid fluid overload. We used laminaria Woodside, Australia for dilation of the cervix 6 hours before surgery. In some patients, cervix was dilated using Hegar No 9 (Martin Hegar, Germany) before hysteroscopy.

### Metroplasty

Septum incision was made using a resectoscope loop with setting at 60W for cutting. After the identification of tubal ostia septum incision was performed until reaching the typical pink tissue which was underlying myometrial tissue, but it didn’t enter the myometrium in order to avoid severe bleeding. The incision was performed until there was no residual septum between the tubal os. This procedure also shows the proximity of the uterine serosa assessed by laparoscopy.

### Myomectomy and polypectomy

The procedure was performed using a 27 French wire monopolar-cutting loop. The first step of intervention was to evaluate the uterine cavity and the characteristic of the myomas or polyp. The intera-cavity component was removed by repeated passage of the monopolar angled cutting loop using the slicing technique. All procedures were videotaped, and all resected specimens were sent for histological analysis.

### Adhesiolysis

We used hydrodissection for thin or filmy adhesions (grade I), whereas for other grads (II, III) of adhesions, we used needle electrode, loop electrode or scissor under direct vision. The cutting and coagulating power was set at 60 W. At the end of procedure, hormone treatment was started consisting of conjugated estrogen 1.25 mg daily for 25 days with the addition of Medroxy progesterone acetate (Iran Hormone, Iran) at a dose of 10 mg per day in the last ten days of the cycle. This treatment continued for 2 months.

We evaluated the reproductive outcomes, early and late complications of each patient, during a period of 1 year after surgery. According to patients, chief complaint success was determined by relief of symptoms. Failure was recorded if subsequent intervention was required or symptoms persisted.

Statistical analysis was performed using Chi-squared (χ²) test and descriptive test. The Statistical Package for Social Sciences (SPSS; SPSS Inc., Chicago, IL, USA) version 11, and a value of p<0.05 was considered as statistically significant.

## Results

Chief complaint, pathology, parity and type of infertility are summarized in table 1, while the obtained results showed that abnormal uterine bleeding was the most frequent chief complain in infertile women. The mean age of the patients was 31.49 ± 5.7 years, with the minimum and the maximum of 20 and 44 years old, respectively.

Before hysteroscopy, our multigravida patients had 108 case of pregnancy, of which 87 ended in abortion (80.55%). After hysteroscopy metroplasty, 13 patients had pregnancy (65%), of which 4 had abortion and 9 had term pregnancy (45%). Among patients with recurrent abortion, 6 patients (75%) completed their pregnancy, successfully. Pregnancy rate after myomectomy was 25% (4 out of 16 patients). Although abnormal uterine bleeding relief was found in 72% (17 out of 27 patients), but pregnancy rate was low (Table 2). Chief complaint and treatment result are summarized in table 2.

Fever was shown to be the early complications in 2 patients. Unfortunately, oophorectomy due to ovarian obsess in one patient and hysterectomy due to sever vaginal bleeding in another patient were considered to be the late complications.

The difference between uterine pathology and treatment result was evaluated by Chi-square (χ²) test and shown in table 3. Distribution of chief complaint according to proven pathology in hysteroscopy is shown in table 4. Distribution of participants according to type of infertility has shown in table 5.

**Table 1:** Demographic characteristic, main pathology and chief complaints


**Age, mean ± SD**	31.49 ± 5.7 (Y)
Min	20
Max	44
**Parity**	
Nulliparous	20
Pluriparous	45
**Pathology**	
Septum	20
Myoma	16
Polyp	13
Adhesion	16
**Chief complaints**	
AUB + infertility	27
Abortion + infertility	14
Pure infertility	24
**Type of infertility**	
Primary	20
Secondary	37
Recurrent abortion	8

AUB; Abnormal uterine bleeding.

**Table 2:** Distribution of treatment results according to chief complaints.


Pathology Chief complain	No relief	Term pregnancy	Abortion	Relief of symptoms	Statistical test^*^

**AUB+infertility**	4 14.8%	5 18.5%	1 3.7%	17 62.9%	χ²=32.4 p<0.001
**Abortion+ infertility**	5 37.5%	8 57.1%	1 7.1%	0	χ²=7.3 p=0.06
**Pure infertility**	10 41.7%	10 41.7%	4 16.7%	0	χ²=14.6 p=0.002


*; Each pathology was compared with rest of study population (df=3) and AUB; Abnormal uterine bleeding.

**Table 3:** Distribution of treatment results according to uterine pathology procedure.


Result Pathology	No relief	Positive pregnancy test	Term pregnancy	Abortion	Relief the symptoms	Statistical test^*^

**Septum**	7(35%)	13(65%)	9(45%)	4(20%)	0	χ²=13.1 p=0.010
**Myomas**	3(18.8%)	4(25%)	4(25%)	0	9(56.3%)	χ²=13.3 p=0.009
**Polyp**	2(15.4%)	6(46%)	6(46.2%)	0	5(38.5%)	χ²=4.01 p=0.404
**Adhesion**	7(43.8%)	6(37.5%)	4(25%)	2(12.5%)	3(26.2%)	χ²=13.1 p=0.534


*; Each pathology was compared with rest of study population (df=4). Positive pregnancy rate was successful rate of our procedure achieved after inducing a term pregnancy according to anatomical pathology and the efficacy of surgery.

**Table 4:** Distribution of chief complaints according to pathology results in hysteroscopy procedure


PathologyChief complaints	Septum	Myoma	Polyp	Adhesion

**AUB+ infertility**	0	14	7	6
** Abortion+infertility**	12	0	1	1
**Pure infertility**	8	2	5	9


AUB; Abnormal uterine bleeding.

**Table 5:** Distribution of participants according to type of infertility


ResultInfertility	No relief	Term pregnancy	Abortion	Relief of symptoms

**Primary**	9(45%)	6(30%)	4(20%)	1(5%)
**Secondary**	8(21.8%)	11(29.7%)	2(5%)	16(43.2%)
**Recurrent abortion**	2(25%)	6(75%)	-	-


P value; 0.00.

## Discussion

Structural uterine lesions has been assumed to cause infertility, while several studies have shown very poor reproductive performance with high miscarriage and low term pregnancy rates when malformation is not treated. Septate uterus is considered to be one of these malformations and many studies have reported a significant improvement of pregnancy outcome after incision of the septum (5, 7). In our study, pregnancy rate after metroplasty was 65%, of which more than 70 percent achieved live birth. Among 9 full-term pregnancies in patients with septate uterus, most pregnancies occurred in patients with recurrent abortion rather than infertility. It seems that outcome of patients with miscarriages is better than the patients with infertility, as mentioned in other studies (4, 8).

Polypectomy had the best obstetrical outcome in our study and supported this idea that small intrauterine lesions such polyps causing implantation failure show better results after polypectomy (2, 9). More than 70% of patients with polyp pathology and abnormal uterine bleeding cured after hysteroscopy polypectomy. As mentioned in Lasmar’s study, endometrial polyp is the most frequent hysteroscopic finding in patient with abnormal uterine bleeding who responds well to this treatment (6).

After adhesiolysis, more than 60 percent of patients had improvement in menstruation. Although pregnancy rate was 37%, but others found normal menstrual pattern after hysteroscopic adhesiolysis. Our findings are differentwith others research (10) and this may be due to small number of patients.

Abnormal uterine bleeding (AUB) and reproductive problems are the most common indications for hysteroscopic myomectomy (11, 12). In our study, AUB was the main chief complaint before myomectomy, and more than 50% relieved after surgery, but reproductive outcome was poor. It seems that endometrial damage and intrauterine adhesions after hysteroscopic myomectomy have potential adverse effects on their fertility as mentioned in other studies (13, 14).

So, further studies is needed to determine the relationship between AUB and infertility. Sample size was small in our study. Controlled study with large sample size is needed in order to achieve better results.

## Conclusion

We have best result in septoplasty and hysteroscopic polypectomy. Hysteroscopic adhesiolysis and myomectomy had poor outcomes due to the nature of pathology.

## References

[1] Homer HA, Li TC, Cook ID (2000). The septate uterus: a review of management and reproductive outcome. Fertil Steril.

[2] Cicinelli E, Matteo M, Causio F, Schonauer LM, Pinto V, Galantino P (2001). Tolerability of the mini-pan-endoscopic approach (transvaginal hydrolaparascopy and minihysteroscopy) versus hysterosalpingography in an outpatient infertility investigation. Fertil Steril.

[3] Ventolini G, Zhang M, Gruber J (2004). Hysteroscopy in the evaluation of patients with recurrent pregnancy loss: a cohort study in a primary care population. Surg Endosc.

[4] Saygili-Yilmaz E, Yildiz S, Erman-Akar M, Akyuze G, Yilmaze Z (2003). Reproductive outcome of septate uteruse after hysteroscopic metroplasty. Arch Gynecol Obstet.

[5] Valli E, Vaquero E, Lazzarin N, Castera D, Marconi D, Zupi E (2004). Hysteroscopic metroplasty improves gestational outcome in women with recurrent spontaneous abortion. J Am Assoc Gynecol Laparosc.

[6] Lasmar RB, Dias R, Barrozo PR, Oliveira MA, Coutinho Eda S, da Rosa DB (2008). Prevalence of hysteroscopic finding and histologic diagnoses in patients with abnormal uterine bleeding. Fertil steril.

[7] Porcu G, Cravello L, D'Ercole C, Cohen D, Roger V, De Montgolfier R (2000). Hysteroscopic metroplasty for septate uterus and repetitive abortion: reproductive outcome. Eur J Obsetet Gynecol Reprod Biol.

[8] Saygili-Yilmaz ES, Erman-Akar M, Yilmaz Z (2002). A retrospective study on the reproductive outcome of the septate uterus corrected by hysteroscopic metroplasty. Int J Gynaecol Obstet.

[9] Rama Raju GA, Shashi Kumari G, Krishna KM, Prakash GJ, Madan K (2006). Assessment of uterine cavity by hysteroscopy in assisted reproduction programme and its influence on pregnancy outcome. Arch Gynecol Obstet.

[10] Yu D, Li TC, Xia E, Huang X, Liu Y, Peng X (2008). Factors affecting reproductive outcome of hysteroscopic adhesiolysis for Asherman’s syndrome. Fertil Steril.

[11] Camanni M, Bonino L, Delpiano EM, Ferrero B, Migliaretti G, Deltetto F (2010). Hysteroscopic management of large symptomatic submucous uterine myomas. J Minim Invasive Gynecol.

[12] Somigliana E, Vercellini P, Daguati R (2007). Fibroids and female reproduction: a critical analysis of the evidence. Hum Reprod Update.

[13] Yen CF, Lee CL, Wang CJ, Soong YK, Arici A (2007). Successful pregnancies in women with diffuse uterine leiomyomatosis after hysteroscopic management. Fertil Steril.

[14] Shokeir T, El-Shafei M, Yusef H, Allam AF, Sadek E (2010). Submucuos myoma and their implications in the pregnancy rates of patients with otherwise unexplained primary infertility undergoing hysteroscopic myomectomy: a randomized matched control study. Fertil Steril.

